# The Interplay of Adolescents’ Aggression and Victimization with Friendship and Antipathy Networks within an Educational Prosocial Intervention

**DOI:** 10.1007/s10964-019-01105-z

**Published:** 2019-09-03

**Authors:** Diego Palacios, Christian Berger, Bernadette Paula Luengo Kanacri, René Veenstra, Jan Kornelis Dijkstra

**Affiliations:** 1 Department of Sociology, Interuniversity Center for Social Science Theory and Methodology (ICS), University of Groningen, Grote Rozenstraat 31, 9712 TG Groningen, The Netherlands; 2 Department of Psychology, Pontificia Universidad Católica de Chile, Santiago, Chile

**Keywords:** Social network analysis, Prosocial intervention, Friendship, Antipathies, RSiena, Dyadic perception

## Abstract

How the interplay between peer relationships and behaviors unfolds and how this differs between classrooms is an understudied topic. This study examined whether adolescents befriend or dislike peers whom they consider as aggressor or victim and whether these results differ in classrooms that received an intervention to promote prosocial behavior compared to classrooms without the intervention. The sample was composed of 659 seventh graders (*M*_*age*_ = 12.32; 48% girls) from nine intervention and seven control classrooms in eight schools in Santiago, Chile. It was hypothesized that adolescents in intervention classrooms would be less befriended and more disliked by classmates who considered them as aggressors, and more befriended and less disliked by classmates who considered them as victims, compared to control classrooms. Longitudinal multiplex social network analyses (RSiena) indicate that antipathies toward peers considered as aggressive and victimized were significantly lower in intervention classrooms than in control classrooms, but no significant differences were found for friendships. ﻿These findings suggest that the impact of an educational intervention may go beyond changing individual behavior and extend to the way peer relations develop in classrooms.

## Introduction

Peers constitute an important social context for adolescents’ development (Furman and Rose 2015). Peer relations may take positive forms, such as friendships (Bagwell and Smith 2011), but also negative forms, such as antipathies (Berger et al. 2011; Card 2010). Both types of relations have been linked to aggression and victimization. The detrimental role of aggression in the emergence and maintenance of friendships and antipathies in adolescence has been widely reported. Research indicate that aggressive youth are less likely to be selected as friends (e.g., Logis et al. 2013). Similarly, adolescents who display aggressive behavior are commonly disliked by peers (Card and Hodges 2007; van den Broek et al. 2016). Victimization also plays a role in the formation and maintenance of friendships and antipathies. Adolescents who are victimized tend to be socially isolated and have fewer friends (Berger and Rodkin 2009). In fact, if victims do not have friends, they might end up isolated and disliked by their peers (Salmivalli et al. 2000; Scholte et al. 2009), and continue to be victimized (Sentse et al. 2017).

Peer relationships do not emerge in isolation but arise in the larger peer context. As students spend a large part of their time interacting with classmates, classrooms are important in adolescents’ social development (Card and Schwartz 2009). Classrooms might, however, differ in the way behaviors are evaluated and appreciated (Dijkstra and Gest 2015), and therefore differ in promoting prosocial and nurturing relationships (Schacter and Juvonen 2018) or, by contrast, in fostering negative peer processes, such as rejection and victimization (Berger and Caravita 2016; Babarro et al. 2017). Social norms that sanction aggression, or promote and value prosocial behaviors, are relevant for interpersonal processes and might play a central role in how the perception of aggression and victimization affect peer relations such as friendships and antipathies. One way to change social norms is via educational interventions that can promote classroom peer ecologies in which adolescents positively regulate their behaviors improving mutual prosocial responses, cooperation and supportiveness, thereby creating a naturally positive and more inclusive classroom environment (Caprara et al. 2015; Luengo Kanacri et al. 2017).

This study aims to examine whether an educational intervention impacts the association between the adolescents’ perception of peers’ aggression and victimization, and friendship and antipathy relationships ﻿by adopting a longitudinal social network approach. In order to do this, classrooms participating in an educational intervention aimed at promoting prosocial behavior and social cohesion, ProCiviCo (*Promoting prosocial behavior and civic engagement for social cohesion in school settings*; Luengo Kanacri and Jiménez-Moya 2017) were compared with control classrooms. This study incorporates a novel perspective by examining the dyadic perception (student A’s perception of student B’s behavior) about aggression and victimization as network information. This approach allows assessing the effect of perceiving a peer as aggressive or victimized on the interpersonal relationships with that adolescent, either positive (friendship) or negative (antipathy). It is expected that the interplay between the dyadic perceptions of aggression and victimization, and friendships and antipathies would differ between the intervention and control classrooms due to differences in peer norms and normative behaviors.

### Aggression, Friendships and Antipathies

Studies have consistently shown that befriended adolescents display similar levels of aggressive behavior (Dijkstra et al. 2011), although possibly based on a default selection in which aggressive adolescents are left with similar peers as the only option for establishing friendships (Deptula and Cohen 2004; Sijtsema and Lindenberg 2018). This default selection builds on studies showing that aggressive adolescents are less likely to be selected as friends (Logis et al. 2013), although they are usually nominated as cool and popular. This implies that aggression is a valued social asset, as shown by several studies evidencing its association with popularity and coolness (Berger and Rodkin 2012; Kiefer and Wang 2016). However, aggression is also a rejected attribute (Ettekal and Ladd 2015). Although aggressive adolescents are popular and cool, they are not socially preferred (Kraft and Mayeux 2018), which might explain their lower friendship’ nomination rates. For instance, several studies show that adolescents who bully are disliked (Pouwels et al. 2016; van den Broek et al. 2016), probably because it generates anxiety and fear (Vaillancourt et al. 2010).

### Victimization, Friendships and Antipathies

Adolescents who experience peer victimization tend to have fewer friends (Berger et al. 2019). Peers avoid befriending victimized adolescents because of fear of becoming victimized themselves (Boulton 2013). Having fewer friendships represents a social disadvantage for victimized adolescents because friendships are important for social adaptation and well-being (Holder and Coleman 2015; Lansford et al. 2014). Friends can offer support and protection when necessary (Cuadros and Berger 2016), but also enable adolescents to build and confirm their identities (Bukowski and Sippola 2005). Conversely, if victims do not have friends, they might end up isolated and disliked by their peers (Salmivalli et al. 2000; Scholte et al. 2009), and continue to be victimized (Sentse et al. 2017). Although previous studies show that rejection can lead to peer victimization (Salmivalli and Isaacs 2005; Serdiouk et al. 2015), the path from being victimized to being rejected has been less studied.

### Peer Relationships within Educational Contexts

Schools are important socializing venues for promoting prosocial behavior and civic engagement. Educational interventions following a Socioemotional Learning (SEL) framework (Durlak et al. 2015), besides having a direct effect on individual behavior, also have an impact on school social climate. For instance, Hendrickx et al. (2016) showed that when students perceived higher teacher support, the classroom peer ecology was more prosocial and rejection rates were lower. Seemingly, classrooms’ prosocial norms (both descriptive and prescriptive) were associated with higher levels of individual prosocial behavior (Laninga-Wijnen et al. 2018a). Interventions focusing on behaviors involving cooperation, helping, sharing, and displaying concern for others (Eisenberg et al. 2006) may be effective strategies to produce more positive, cooperative social interactions (Batson 2011) and to reduce both the emergence and the negative consequences of aggression and victimization (Obsuth et al. 2015). In this sense, educational interventions such as ProCiviCo could foster classroom peer ecologies in which adolescents positively regulate their behaviors improving mutual prosocial responses, cooperation and supportiveness, producing a positive and more inclusive classroom environment (Caprara et al. 2015; Luengo Kanacri et al. 2017). It is expected that in positive environments, adolescents that are responsive to peers’ problems and difficulties and are able to help them would be supportive to victims in terms of befriending them more frequently and rejecting them less frequently. Conversely, because the adoption of prosocial norms and the development of prosocial behavior are to a greater extent considered as incompatible with aggressive behavior (Siu et al. 2012), adolescents who display aggressive behaviors would be negatively sanctioned by means of not befriending and rejecting them more frequently.

### The Effect of Prosocial Behavior and Sex

The literature on peer relations shows that adolescents who display prosocial behaviors are valued as friends (Poorthuis et al. 2012) and are socially preferred by their peers (Berger et al. 2015; Card 2010). Moreover, several studies report a negative association of prosocial behavior with both aggression (Berger et al. 2015; Molano et al. 2013) and victimization (Coleman and Byrd 2003; Griese et al. 2016). Because the focus of ProCiviCo was the promotion of prosocial behavior among peers, this intervention should also affect friendships, antipathies, and perceptions of peers’ aggression and victimization. Therefore, individual levels of prosocial behavior need to be controlled for.

Seemingly, there is ample evidence on the effects of sex on friendships, particularly a preference for same-sex over cross-sex friendships during adolescence (Simpkins et al. 2013; Veenstra and Dijkstra 2011). Conversely, the evidence about same-sex antipathies (Rambaran et al. 2015; Witkow et al. 2005) and sex differences in aggression is still inconclusive (Batanova and Loukas 2011; Peets and Kikas 2006). For instance, Faris and Felmlee (2011) found that differences in aggression are less attributable to individual sex differences, and are more dependent on social ecology and in particular the implications of aggression for social status. Similarly, earlier studies show sex differences in peer victimization, both in their frequency and implications (Berger and Rodkin 2009), which again might suggest differential experiences of victimization for boys and girls. Thus, sex should also be taken into account when studying peer processes (Sentse et al. 2015).

## Current Study

**Fig. 1:**
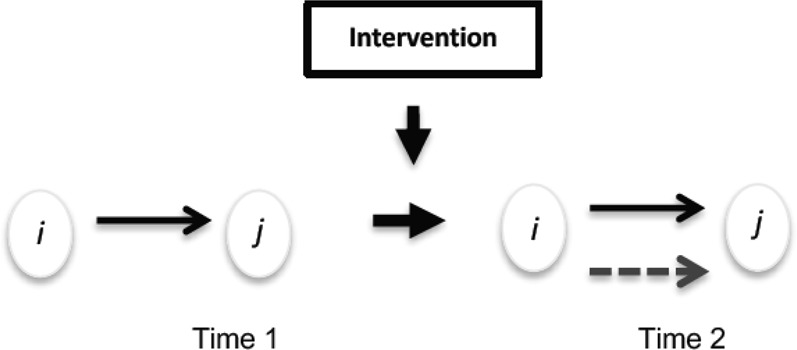
The figure represents whether an existing tie from student *i* to *j* in one type of network (e.g., aggression, victimization) leads to the formation or maintenance of a tie in another type of network (e.g., friendship, antipathy), moderated by receiving the intervention

The present study examines the extent to which the dyadic perceptions of peers’ aggression and victimization are related to friendships and antipathies (see Fig. 1) comparing network processes in intervention and control classrooms using longitudinal multiplex social network analysis (Snijders et al. 2013). To this end, the perception of peers’ aggression and victimization, along with friendships and antipathies, are treated as network relationships, examining the associations between the dyadic perception of peer’s aggression and victimization, and friendship and antipathy relationships. It is expected that in intervention classrooms (characterized by higher levels of cooperation, empathy, and concern for others), compared to control classrooms, students would be less likely to exclude victimized adolescents, but not aggressors, by befriending them. Consequently, compared to control classrooms, adolescents in the intervention classrooms would be less befriended by classmates who consider them as aggressors (Hypothesis 1) and more befriended by classmates who consider them as victims (Hypothesis 2). Furthermore, positive classroom environments would be particularly relevant for those who are generally more disliked, such as aggressive peers and victimized adolescents. Accordingly, compared to control classrooms, adolescents in the intervention classrooms would be more disliked by classmates who consider them as aggressors (Hypothesis 3) and less disliked by classmates who consider them as victims (Hypothesis 4). Moreover, considering the relevance of both prosocial behavior and sex on peer relations (friendships and antipathies) as well as on aggression and victimization, the analyses controlled for individual effects of prosocial behavior and sex.

## Method

This study is part of a larger project aimed at developing, implementing and evaluating a school-based intervention to promote prosocial behavior and civic engagement, *ProCiviCo* (Luengo Kanacri and Jiménez-Moya 2017). The intervention as designed and implemented in Chile was adapted from an intervention created in Italy (CEPIDEA) and also implemented in Colombia (Caprara et al. 2015). The intervention is intended to promote interpersonal social cohesion among students by increasing adolescents’ prosocial behavior and civic engagement and its main determinants, referring to emotion regulation, empathic skills, prosocial moral values, (Luengo Kanacri et al. 2014). The program includes five components: (a) prosocial responding in the peer context, (b) empathic skills, (c) emotion regulation, (d) prejudice and social identities, (e) and civic engagement within the school community. The intervention used two main strategies over an academic year: workshops and lessons. Workshops were led by the research team, but in collaboration with the teachers, and consisted of weekly group discussions, role-playing, and interviews. Lessons were led by teachers and consisted of integrating civic issues in regular classwork across subjects. On average, the number of workshops was 16 per school and 4–5 lessons per classroom. The intervention is centered around the idea that prosocial behavior, as an exercise of active citizenship, can be taught and developed through appropriate formative experiences (for details about the intervention see Luengo Kanacri and Jiménez-Moya 2017; Luengo Kanacri et al. 2019). A cluster randomized controlled trial of the ProCiviCo program showed positive effects on prosocial behavior across multiple informants (students, parents, and teachers) which in turn decreased aggressive behaviors among adolescents (Luengo Kanacri et al. 2019).

### Sample

Initially, the data was composed of 659 seventh graders from Santiago (Chile) from 16 classrooms (*M*_*age*_ = 12.32; *SD=* 0.22, 48% girls) from eight public and private subsidized schools. Schools were randomly assigned to the intervention (nine classrooms from four schools) and control (seven classrooms from four schools) condition. According to the Chilean Ministry of Education, these schools are considered as middle-low to middle socioeconomic status schools. The average classroom size was 41.2 students (*SD=* 8.1, range from 29 to 51). The intervention ran from May till November 2017. Students were measured three times over the study: pre-test (April 2017), post-test (November 2017), and a follow-up assessment (May 2018). All participants attended seventh grade at the pre-test.

Three classrooms were excluded from the analyses. First, an only-boy classroom was excluded because of potential different processes regarding aggression and social norms in single sex-classrooms (Johnson and Gastic 2014). A second classroom was excluded because of its combination of a few tie changes between assessments, a small fraction of stable relationships relative to all new, lost, and stable relationships, and a high percentage of missing data (for details see Appendix A1). Finally, ﻿due to some convergence issues in the social network analyses (i.e., low reliability of estimates), a third classroom was excluded. The final sample contained 530 students from seven intervention (*M*_*age t1*_ = 12.35; *SD* = 0.21, % 47 girls) and six control classrooms (*M*_*age t1*_ = 12.29, *SD* = 0.26; 61% girls).

Students in Chilean schools tend to remain together with their classmates across elementary education (first to eighth grade). Therefore, classrooms are stable environments in which peer relations unfold. Despite this particularity, research on adolescent peer relations with Chilean samples has shown similar patterns to American and European populations (Berger and Rodkin 2012; Dijkstra et al. 2011), and the study on peer relations and adolescent development in Latin America follows similar trends to those in western societies (Berger et al. 2016).

### Procedure

Questionnaires were administered to the whole classroom in regular school hours in the presence of research assistants. Children were assured that their answers would be kept confidential and that they could stop participating at any time. Measures and procedures to protect the confidentiality and rights of participants were approved by the Institutional Review Board of the participating university. Parental active consent and adolescents’ assent were obtained for all participants included in the study.

### Measures

Peer nominations procedures assessed aggression, victimization, friendships, and antipathies (Cillessen and Mayeux 2004). Participants were asked to check on a roster and nominate up to three classmates per measure. Adjacency matrices were created for each classroom on each assessment, representing the different networks with nominations coded as 1 and non-nominations coded as 0.

#### Aggression networks (T1–T3)

A comprehensive measure of aggression was used (Hamre and Pianta 2006; Logis et al. 2013). Participants were asked to identify classmates who best fit the descriptor *they behave aggressively or make fun of others* (average degree_t1_ = 2.47, *SD*_t1_ = 0.37; average degree_t2_ = 2.54, *SD*_t2_ = 0.32; average degree_t3_ = 2.27, *SD*_t3_ = 0.25).

#### Victimization networks (T1–T3)

Participants were asked to identify classmates who best fit the descriptor *they are victimized, or kids make fun of him* (Dijkstra et al. 2010; average degree_t1_ = 2.37, *SD*_t1_ = 0.37; average degree_t2_ = 2.47, *SD*_t2_ = 0.36; average degree_t3_ = 2.17, *SD*_t3_ = 0.27).

#### Friendship networks (T1–T3)

Participants were asked to identify classmates who best fit the descriptor *with whom do you hang out at school during recess* (Espelage et al. 2003; Schacter et al. 2014; average degree_t1_ = 2.51, *SD*_t1_ = 0.35; average degree_t2_ = 2.54, *SD*_t2_ = 0.36; average degree_t3_ = 2.32, *SD*_t3_ = 0.27).

#### Antipathy networks (T1–T3)

Participants were asked to identify classmates who best fit the descriptor *with whom would you not like to hang out at school during recess* (average degree_t1_ = 2.55, *SD*_t1_ = 0.38; average degree_t2_ = 2.55, *SD*_t2_ = 0.34; average degree_t3_ = 2.27, *SD*_t3_ = 0.29).

#### Prosocial behavior (T1–T2)

Students rated their own prosocial behavior using the 16-item Prosociality Scale (Caprara et al. 2005). Sample items are *“I am available for volunteer activities to help those who are in need”, “I try to help others*, and *I am emphatic with those who are in need*”. Each item was rated on a 5-point scale from 1 = *(almost) never true* to 5 = (*almost) always true* (*M*_t1_ = 3.48, *SD*_t1_ = 0.16, *M*_t2_ = 3.43, *SD*_t2_ = 0.19; α_t1_ = 0.90, α_t2_ = 0.91).

#### Sex

Participants were asked about their sex, which was coded 0 for boys and 1 for girls (for details see Appendix A2).

### Analytic Strategy

Analyses were conducted using longitudinal social network modeling (RSiena; Simulation Investigation for Empirical Network Analysis). This allowed us to unravel the development of aggression, victimization, friendship, and antipathy networks over time (Ripley et al. 2018) while taking into account network structural effects (e.g., reciprocity, transitivity) as well as students’ individual covariates (e.g., sex and prosocial behavior). RSiena models are actor-based models (Snijders et al. 2010), which assume that actors (here; students) modify their relationships (here; aggression, victimization, friendships and antipathies) between assessments based on their individual preferences. The model determines likely trajectories between observations with the information from time 1 taken as a starting point. The estimates of the model are obtained through an iterative simulation following a Markov Chain Monte Carlo approach (Burk et al. 2007) expressing the strength of the effects included in the model. These unstandardized estimates are comparable to regression coefficients in (logistic) regression indicating the importance of each effect (predictor variables) in creating or maintaining a tie. Missing data due to non-response were handled through the RSiena default missing data method, and participants who joined and left the classrooms network in-between time points were treated using structural zeros (for details see Appendix B1).

The model was estimated for each classroom separately using the Methods of Moments estimator and specifying 5000 iterations in phase 3 for calculating standard errors. To test the four hypotheses and to keep the model parsimonious, two models were estimated: The first including friendship, aggression, and victimization networks (hereafter referred to as the friendship model), and the second one including antipathies, aggression, and victimization networks (hereafter referred to as the antipathy model). For each model (friendship and antipathy), two separate meta-analyses were conducted: the first for intervention classrooms and the second for control classrooms (for more details, see Appendix B2). After that, test statistics^1^were performed to examine significant differences between the parameter estimates related to the hypotheses. Finally, to help the interpretation and comparison between intervention and control classrooms, the expected relative importance of each effect was calculated for each classroom and then averaged for intervention and control classrooms (Indlekofer and Brandes 2013). This measure is analogous to an effect size measure capturing the influence of each effect on actor’s decisions of creating or maintaining ties. The sum of the expected relative importance of all effects included in a model is 1.

### Model Selection Procedure

The choice of the model parameters was based on recent research that used multiplex social networks analyses (Huitsing et al. 2012, 2014; Rambaran et al. 2015) as well as research on friendship and antipathy networks (Berger and Dijkstra 2013) (for details see Appendix B3). Moreover, time heterogeneity tests indicated no significant differences between effects’ estimates across periods for most classrooms (for details see Appendix B4). Accordingly, the information from the two periods (from time 1 to 2, and from time 2 to 3) was examined in one model. Also, goodness of fit tests were conducted to assess how well the model reproduced auxiliary network statistics (outdegree, indegree, geodesic distance, and triad census distributions) of the observed data not explicitly fit in the model (Lospinoso 2012). Overall, the results for the four types of networks indicated an excellent representation of the indegree, geodesic distance, and triad census distributions, and an acceptable representation of the outdegree distribution (for details see Appendix B5).

### Model Specification

#### Structural network effects

These effects were included to capture the basic tendencies of actors to form and maintain relationships within the different types of networks. *Density* describes the tendency of actors to establish relationships. *Reciprocity* is the tendency to reciprocate relationships (referring to forming mutual ties). Only for friendship networks, two versions of the geometrically weighted edgewise shared partners (GWESP) were included: one to measure the tendency of students to become friends with the friends of their friends (*transitivity* GWESP FF), and other to capture the tendency toward non-hierarchical triadic structures (*cyclical* GWESP BB). For the four types of networks, the *indegree-popularity*, and *indegree-activity* effects were included representing the tendency of actors who receive many nominations to receive and to send more nominations over time, respectively. Finally, to improve the goodness of fit of the models, the *balance* effect was added, representing the similarity between the outgoing ties of student *i* and the outgoing ties of the other students *j* to whom *i* is tied, indicating the preference for classmates who choose the same as *i*. Because aggression and victimization were measured as perception networks, the reciprocity and triadic effects for both types of networks were not included.

#### Covariates

Sex and prosocial behavior were included as control variables, by including the selection effects for each of these covariates. These selection effects can be either dynamic (referring to change over time) or remain constant. Three selection dynamic effects (*prosocial behavior alter, prosocial behavior ego, prosocial behavior similarity*) and three selection constant effects (*sex alter, sex ego, same-sex*) were included. The alter and ego effects capture the effects of covariates on received nominations (“popularity” effect) or given nominations (“activity” effect), respectively. The same and similarity effects capture the effect of similarity for covariates on tie formation or maintenance between a focal actor (ego) and a peer (alter).

#### Cross-network effects

For the four types of networks, the entrainment effect was included, referring to the extent to which the existence of a tie from the student *i to j* promotes the creation or maintenance of a tie in another type of network from the student *i* to *j*. The four hypotheses were tested through the effect of aggression and victimization ties on friendship ties (Hypotheses 1 and 2), and the effect of aggression and victimization ties on antipathies ties (Hypotheses 3 and 4), controlling for the four opposite effects (referring to the effect of friendships on aggression and victimization ties, and the effect of antipathies on aggression and victimization ties).

## Results

### Descriptive Analysis

Table 1 provides descriptive information about the changes in the four types of networks from time 1 to 2 (period 1), and from time 2 to 3 (period 2). Distance shows that the number of ties changes was higher in the first period than in the second period. Similarly, Jaccard indexes (referring to tie stability between two consecutive assessments) indicate a substantial rearrangement of ties between assessments, with antipathy, aggression, and victimization ties being less stable than friendship ties. In the case of antipathy networks, previous research has shown its stability tend to be above 0.20 (Berger and Dijkstra 2013; Daniel et al. 2016; Rambaran et al. 2015). Also, Jaccard indexes in the first period were slightly higher than in the second period, suggesting an effect of the summer break (January and February in Chile) on classrooms’ composition (referring to students who left classroom at the end of the academic year, and students who joined classrooms at the beginning of the new academic year). Although a Jaccard index of at least 0.20 is recommended for using stochastic actor-oriented models (Ripley et al. 2018), satisfactory convergence was obtained (overall maximum convergence ratios < 0.20 and mean absolute individual *t* statistics < 0.10 for all models).

**Table 1:** Average changes in networks variables across the three observations for intervention and control classrooms

	Intervention classrooms (*n* = 7)		Control classrooms (*n* = 6)	
	T1 → T2	T2 → T3	T1 → T2	T2 → T3
*N* students total	256		274	
Antipathy networks				
Number of tie changes (distance)^a^	117.3	103.6	109.2	95.4
Jacccard index (stability)^b^	0.15	0.13	0.18	0.16
Creating tie (0 → 1)	68.0	62.4	62.5	61.3
Disolving tie (1 → 0)	65.0	71.1	62.2	68.0
Stable tie (1 → 1)	23.0	18.6	26.8	23.4
Friendship networks				
Number of tie changes (distance)	76.9	70.1	81.7	70.3
Jacccard index (stability)	0.35	0.30	0.30	0.27
Creating tie (0 → 1)	47.0	43.6	47.3	45.7
Disolving tie (1 → 0)	42.0	51.3	47.3	51.8
Stable tie (1 → 1)	43.7	39.3	41.3	36.3
Aggression networks				
Number of tie changes (distance)	104.9	91.0	83.8	81.0
Jacccard index (stability)	0.20	0.17	0.27	0.22
Creating tie (0 → 1)	62.6	56.4	52.8	47.6
Disolving tie (1 → 0)	56.0	64.9	49.2	59.0
Stable tie (1 → 1)	28.0	24.0	38.0	31.2
Victimization networks				
Number of tie changes (distance)	104.3	90.7	97.4	84.8
Jacccard index (stability)	0.14	0.14	0.23	0.19
Creating tie (0 → 1)	67.3	57.1	55.6	51.2
Disolving tie (1 → 0)	57.1	65.4	54.2	61.2
Stable tie (1 → 1)	20.9	20.3	32.0	25.8

^a^The Hamming distance reflects the total number of nominations in the network for which there is observed change between data observations and includes the sum of new nominations and lost nomination

^b^Network stability was measured by the Jaccard index which reflects the number of changing relationships between assessments

### Longitudinal Social Networks Analysis

Tables 2 and 3 present the results of the RSiena analyses for the friendship and antipathy models comparing intervention and control classrooms. Because the focus of this study was on the cross-network effects, the results of structural network effects and covariates (sex and prosocial behavior) were reported succinctly.

**Table 2:** Meta-analysis results from longitudinal multiplex models predicting friendship, aggression, and victimization networks

Effects parameters	Intervention classrooms							Control classrooms						
	Est	SE	Σ	Q	RI w1	RI w2	RI w3	Est	SE	Σ	Q	RI w1	RI w2	RI w3
*Friendship*														
Structural effects														
Density	−0.859**	0.224	0.000	3.608	0.11	0.12	0.11	−1.000**	0.230	0.002	2.433	0.17	0.17	0.18
Reciprocity	1.190**	0.294	0.558	12.480	0.08	0.08	0.09	1.009**	0.174	0.000	3.806	0.07	0.08	0.08
Balance	0.261**	0.038	0.000	2.190	0.19	0.21	0.19	0.275**	0.044	0.000	2.895	0.26	0.26	0.25
Transitivity GWESP FF	1.165**	0.316	0.000	5.092	0.12	0.13	0.14	1.137**	0.302	0.000	3.393	0.08	0.09	0.09
Cyclical GWESP BB	0.333	0.258	0.000	2.128	0.05	0.04	0.04	−0.235	0.221	0.000	2.100	0.02	0.03	0.02
Indegree—popularity	−0.053	0.034	0.000	2.903	0.06	0.07	0.06	−0.100*	0.035	0.000	2.306	0.10	0.10	0.10
Indegree—activity	−0.425**	0.080	0.000	1.527	0.15	0.14	0.15	−0.160*	0.071	0.000	0.993	0.05	0.05	0.05
Covariate effects														
Sex (girls) alter	−0.033	0.126	0.209	11.456	0.05	0.05	0.05	−0.143	0.095	0.135	0.000	0.03	0.03	0.03
Sex (girls) ego	0.083	0.112	0.000	2.089	0.01	0.01	0.01	−0.016	0.117	0.894	0.000	0.01	0.01	0.01
Same sex^a^	0.189*	0.089	0.092	6.394	0.06	0.06	0.05	0.338**	0.073	0.000	0.000	0.09	0.08	0.08
Prosocial behavior alter	0.058	0.060	0.000	5.213	0.02	0.02	0.02	0.104	0.075	0.162	0.000	0.02	0.02	0.01
Prosocial behavior sex	0.009	0.089	0.000	4.806	0.02	0.02	0.02	0.018	0.093	0.848	0.000	0.01	0.01	0.01
Prosocial behavior similarity	−0.099	0.230	0.000	3.611	0.02	0.02	0.02	0.279	0.394	0.478	0.681	0.05	0.05	0.05
Cross-network effects														
Aggression to Friendship^a,b^ (Hypothesis 1)	0.090	0.452	0.531	5.135	0.02	0.02	0.01	−0.657	0.480	0.000	0.268	0.02	0.02	0.02
Victimization to Friendship^a^ (Hypothesis 2)	0.016	0.422	0.000	4.058	0.03	0.02	0.02	0.061	0.301	0.000	3.106	0.02	0.02	0.02
Aggression														
Structural effects														
Density	−1.503**	0.100	0.001	6.361	0.40	0.39	0.42	−2.048**	0.266	0.560	21.611*	0.37	0.36	0.39
Balance	0.161**	0.040	0.071	11.073	0.17	0.18	0.15	0.046	0.068	0.131	14.335*	0.12	0.11	0.11
Indegree—popularity	0.082**	0.011	0.020	12.383	0.25	0.25	0.24	0.113**	0.013	0.024	13.980*	0.31	0.34	0.33
Indegree—activity	0.008	0.010	0.000	1.195	0.02	0.02	0.03	0.006	0.008	0.000	1.648	0.02	0.02	0.02
Covariate effects														
Sex (girls) alter	−0.201	0.142	0.339	33.088**	0.08	0.08	0.08	−0.374**	0.094	0.095	6.375	0.08	0.08	0.07
Sex (girls) ego	0.019	0.074	0.000	0.845	0.01	0.01	0.01	−0.044	0.079	0.000	0.163	0.01	0.01	0.01
Prosocial behavior alter	−0.002	0.044	0.022	5.284	0.02	0.02	0.02	0.102	0.062	0.000	1.917	0.02	0.02	0.01
Prosocial behavior sex	−0.038	0.059	0.000	0.856	0.01	0.01	0.01	−0.020	0.063	0.000	1.018	0.01	0.01	0.01
*Cross-network effects*														
Friendship to Aggression	−0.051	0.215	0.296	7.157	0.03	0.02	0.03	−0.089	0.273	0.000	2.913	0.02	0.02	0.02
Victimization to Aggression	0.437*	0.165	0.000	3.134	0.03	0.02	0.03	0.579*	0.176	0.001	5.514	0.04	0.04	0.03
Victimization														
Structural effects														
Density	−1.503**	0.094	0.000	3.083	0.38	0.38	0.38	−1.561**	0.196	0.392	16.443*	0.36	0.37	0.38
Balance	0.152**	0.041	0.073	11.171	0.18	0.17	0.16	0.141	0.079	0.166	21.579*	0.21	0.22	0.22
Indegree—popularity	0.085**	0.009	0.012	7.378	0.21	0.23	0.23	0.087**	0.012	0.022	13.328*	0.26	0.26	0.23
Indegree—activity	−0.005	0.012	0.000	2.854	0.03	0.04	0.05	0.003	0.009	0.000	0.939	0.01	0.01	0.01
Covariate effects														
Sex (girls) alter	−0.207	0.117	0.267	28.699**	0.07	0.07	0.07	−0.200*	0.069	0.002	5.071	0.06	0.06	0.06
Sex (girls) ego	−0.012	0.073	0.000	1.921	0.02	0.02	0.02	−0.054	0.073	0.000	1.002	0.01	0.01	0.02
Prosocial behavior alter	−0.032	0.039	0.001	6.758	0.03	0.03	0.03	−0.050	0.051	0.000	1.988	0.02	0.02	0.02
Prosocial behavior sex	0.016	0.060	0.000	3.833	0.02	0.02	0.02	−0.025	0.055	0.000	0.824	0.01	0.01	0.01
Cross-network effects														
Friendship to Victimization	0.177	0.147	0.000	4.954	0.03	0.02	0.02	−0.201	0.225	0.000	2.109	0.02	0.02	0.02
Aggression to Victimization	−0.002	0.170	0.000	1.811	0.02	0.02	0.02	0.071	0.191	0.236	5.635	0.02	0.02	0.02

*Σ* standard deviation, *Q* chi-squared test statistic, *RI* expected relative importance effects

*p  < .05; **p < .001

^a^For one intervention classroom these effects were fixed to the average of the rest of the classrooms because of their high standards errors

^b^For two control classrooms these effects were fixed to the average of the rest of the classrooms because of their high standards errors

**Table 3:** Meta-analysis results from longitudinal multiplex models predicting antipathy, aggression, and victimization networks

Effects parameters	Intervention classrooms							Control classrooms						
	Est	*SE*	*Σ*	*Q*	RI w1	RI w2	RI w3	Est	*SE*	*Σ*	*Q*	RI w1	RI w2	RI w3
Antipathy														
Structural effects														
Density	−1.320**	0.105	0.000	3.322	0.37	0.39	0.40	−1.799**	0.211	0.435	19.366*	0.36	0.38	0.40
Reciprocity	0.316*	0.103	0.000	4.694	0.02	0.03	0.03	0.395*	0.137	0.042	6.181	0.03	0.03	0.03
Balance	0.124*	0.048	0.108	27.408**	0.16	0.16	0.15	0.037	0.048	0.092	14.211*	0.13	0.13	0.13
Indegree—popularity	0.061**	0.010	0.000	5.280	0.14	0.14	0.12	0.080**	0.010	0.000	4.809	0.15	0.17	0.15
Indegree—activity	−0.013	0.022	0.019	6.156	0.05	0.05	0.05	−0.016	0.019	0.008	4.018	0.02	0.03	0.03
Covariate effects														
Sex (girls) alter	0.109	0.094	0.189	14.184*	0.05	0.05	0.05	0.053	0.072	0.000	3.630	0.04	0.03	0.03
Sex (girls) ego	0.057	0.073	0.000	0.549	0.01	0.01	0.01	0.033	0.073	0.000	3.262	0.01	0.01	0.01
Same sex	−0.043	0.087	0.189	18.568*	0.06	0.05	0.05	−0.108	0.131	0.276	19.155*	0.06	0.06	0.05
Prosocial behavior alter	−0.018	0.043	0.000	5.542	0.02	0.02	0.02	0.009	0.056	0.047	4.893	0.02	0.02	0.02
Prosocial behavior sex	0.008	0.054	0.000	1.433	0.01	0.01	0.01	−0.028	0.058	0.000	0.819	0.01	0.01	0.01
Prosocial behavior similarity	−0.129	0.142	0.000	1.627	0.02	0.02	0.01	−0.077	0.187	0.000	1.870	0.02	0.02	0.02
Cross-network effects														
Aggression to Antipathy (Hypothesis 3)	0.643**	0.156	0.000	4.172	0.08	0.06	0.06	1.061**	0.181	0.161	4.404	0.11	0.09	0.09
Victimization to Antipathy (Hypothesis 4)	0.100	0.199	0.000	2.336	0.02	0.01	0.02	0.499*	0.163	0.001	6.287	0.04	0.03	0.04
Aggression														
Structural effects														
Density	−1.661**	0.111	0.002	7.316	0.39	0.39	0.43	−2.187**	0.268	0.555	19.491*	0.37	0.37	0.39
Balance	0.159**	0.041	0.074	11.524	0.17	0.17	0.14	0.043	0.065	0.129	14.728*	0.11	0.11	0.11
Indegree—popularity	0.077**	0.011	0.021	12.657*	0.22	0.23	0.21	0.105**	0.011	0.021	11.553*	0.27	0.30	0.29
Indegree—activity	0.009	0.010	0.000	0.961	0.02	0.02	0.03	0.006	0.008	0.000	1.995	0.01	0.02	0.02
Covariate effects														
Sex (girls) alter	−0.235	0.134	0.308	23.178*	0.07	0.07	0.07	−0.448**	0.116	0.171	7.909	0.08	0.08	0.08
Sex (girls) ego	0.022	0.075	0.000	0.452	0.01	0.01	0.01	−0.044	0.082	0.000	0.199	0.01	0.01	0.01
Prosocial behavior alter	0.001	0.047	0.018	5.411	0.02	0.02	0.02	0.087	0.061	0.000	1.572	0.02	0.02	0.01
Prosocial behavior sex	−0.027	0.061	0.000	0.736	0.01	0.01	0.01	−0.035	0.063	0.000	1.172	0.01	0.01	0.01
Cross-network effects														
Antipathy to Aggression	0.813**	0.209	0.232	6.389	0.07	0.05	0.05	1.082**	0.226	0.000	1.381	0.09	0.06	0.06
Victimization to Aggression	0.432*	0.181	0.000	3.980	0.02	0.02	0.02	0.349	0.238	0.298	6.720	0.03	0.03	0.02
Victimization														
Structural effects														
Density	−1.498**	0.102	0.048	5.320	0.35	0.35	0.36	−1.633**	0.200	0.396	16.329*	0.36	0.37	0.38
Balance	0.163**	0.039	0.065	9.408	0.16	0.16	0.15	0.143	0.079	0.165	22.525**	0.20	0.21	0.21
Indegree—popularity	0.085**	0.009	0.010	6.424	0.19	0.21	0.22	0.090**	0.012	0.021	12.213*	0.25	0.25	0.22
Indegree—activity	−0.003	0.012	0.000	2.761	0.02	0.03	0.03	0.005	0.010	0.000	1.349	0.02	0.02	0.01
Covariate effects														
Sex (girls) alter	−0.226	0.128	0.287	23.677*	0.08	0.08	0.08	−0.203*	0.073	0.000	3.420	0.05	0.05	0.05
Sex (girls) ego	−0.004	0.075	0.000	1.983	0.02	0.02	0.02	−0.047	0.077	0.000	0.985	0.01	0.01	0.02
Prosocial behavior alter	−0.039	0.042	0.003	6.315	0.03	0.03	0.03	−0.043	0.052	0.000	1.355	0.02	0.02	0.02
Prosocial behavior sex	0.005	0.065	0.000	3.535	0.02	0.02	0.02	−0.027	0.056	0.000	1.000	0.01	0.01	0.01
Cross-network effects														
Antipathy to Victimization	0.545	0.281	0.002	7.997	0.09	0.07	0.06	0.205	0.377	0.501	7.732	0.06	0.04	0.06
Aggression to Victimization	−0.019	0.243	0.000	4.863	0.05	0.04	0.04	0.005	0.253	0.000	1.905	0.02	0.02	0.02

*Σ* standard deviation, *Q* chi-squared test statistic, *RI* expected relative importance effects

*p< .05; **p< .001

#### Structural network effects

Looking at the structural network effects in intervention and control classrooms revealed similar findings. The negative *density* effect for all type of networks indicates that in all two contexts, participants nominated less than half of their classmates as friends, rejected, aggressive, or victimized students. Also, friendship and antipathy nominations were reciprocal (positive *reciprocity* effect) and tended to be transitive for friendships; that is, friends of friends were likely to become friends (*Transitivity GWESP FF* effect). Moreover, students who received many antipathy, aggression, and victimization nominations tended to receive more nominations in each type of networks over time (a positive *indegree-popularity* effect).

#### Covariates

In both types of classrooms, a significant same-sex preference in selecting friends (*same-sex* Est. _intervention_ = 0.189, *p* < 0.05; Est. _control_ = 0.338, *p* < 0.001) but not in disliking peers were found (*same-sex* Est. _intervention_ = −0.043, *p* < 0.616; Est. _control_ = −0.108, *p* < 0.410). However, the results for the antipathy networks should be interpreted with caution because these parameters are significantly different across the classrooms (*same-sex Q*._intervention_ = 18.568, *Qp* < 0.05; *Q*_control_ = 19.155, *Qp* < 0.05). Also, there were no significant effects of prosocial behavior on friendship or antipathies. Furthermore, regarding the friendship and antipathy model, boys only receive significantly more aggression (*sex* Est._control_ = −0.374, *p* < 0.001; Est._control_ = −0.448, *p* < 0.001) and victimization nominations in control classrooms (Est._control_ = −0.200, *p* < 0.05; Est._control_ = −0.203, *p* < 0.05).

#### Cross-network effects

For the effect of aggression on friendship nominations, there were no significant effects in both types of classrooms (*Aggression to Friendship* Est._intervention_ = 0.090, *p* = 0.842; Est._control_ = −0.657, *p* = 0.171). Moreover, neither a difference between the two effects’ parameters (*z* = 1.132, *p* = 0.128) nor a difference in the expected relative importance for this effect was found (Int_w1_ = 0.02, Int_w2_ = 0.02., Int_w3_ = .01; Con_w1_ = 0.02, Con_w2_ = 02, Con_w3_ = 0.02). These results suggest that there is no relationship between perceiving someone as aggressive and nominating him/her as a friend (not supporting Hypothesis 1). Also, no significant effects were found in both types of classrooms regarding the effect of friendship on aggression nominations (*Friendship to Aggression* Est._intervention_ = −0.051, *p* = 0.812; Est._control_ = −0. 089, *p* = 0.744).

Similarly, no support was found for the second hypothesis as it was no evidence that, first, adolescents were more befriended by classmates who considered them as victims in both types of classrooms (*Victimization to Friendship* Est._intervention_ = 0.016, *p* = 0.969; Est._control_ = 0.061, *p* = 0.839), and second, that a significant difference between the two effects’ parameters (*z* = −0.086, *p* = 0.465) or a difference in the expected relative importance exists (Int_w1_ = 0.03 Int_w2_ = 0.02, Int_w3_ = 0.02; Con_w1_ = 0.02, Con_w2_ = 02, Con_w3_ = 0.02). Additionally, no significant effects in both types of classrooms were found regarding the effect of friendship on victimization nominations (*Friendship to Victimization* Est._intervention_ = 0.177, *p* = 0.227; Est._control_ = −0. 201, *p* = 0.372).

In both intervention and control classrooms, adolescents were more disliked by classmates who considered them as aggressors (*Aggression to Antipathy* Est._intervention_ = 0.643, *p* < 0.001; Est._control_ = 1.061, *p* < 0.001). However, a difference between the two effects’ parameters was found (z = −1.74, *p* < .05), as well as a difference in the expected relative importance for this effect (Int_w1_ = 0.08, Int_w2_ = 0.06, Int_w3_ = 0.06; Con_w1_ = 0.11, Con_w2_ = 0.09, Con_w3_ = 0.09) These results indicate that adolescents who were considered as aggressive were more disliked in control than intervention classrooms, which was in the opposite direction of the third hypothesis. This finding suggests that intervention classrooms could be more inclusive in terms of antipathy nominations even for adolescents considered as aggressive. In addition, in both types of classrooms adolescents who were disliked were also perceived as aggressors (*Antipathies to Aggression* Est._intervention_ = 0.813, *p* < 0.001; Est._control_ = 1.082, *p* < 0.001).

Concerning the effect of victimization on antipathies (fourth hypothesis), adolescents who were perceived as victims were more disliked only in control classrooms (*Victimization to Antipathy* Est._intervention_ = 0.100, *p* = 0.616; Est._control_ = 0.499, *p* < 0.05). The comparison between the parameter estimates (*z* = −1.76, *p* < 0.05) and the expected relative importance of the effects (Int_w1_ = 0.02, Int_w2_ = 0.01, Int_w3_ = 0.02; Con_w1_ = 0.04, Con_w2_ = 0.03, Con_w3_ = 0.04), suggest that victimized adolescents were slightly less disliked by their peers in intervention than in control classrooms (consistent with the fourth hypothesis). In addition, adolescents who were disliked were also perceived as victims in intervention classrooms, although this effect only approached significance (*Antipathies to Victimization* Est._intervention_ = 0.545, *p* = 0.052; Est._control_ = 0.205, *p* = 0.586).

Additionally, and given that the hypotheses were at the classroom (referring to the network) level, it was also possible to confound those effects with mechanisms operating at the individual level. That means that adolescents with higher individual prosocial behavior would more strongly dislike and less strongly befriend whom they consider as aggressors, and less strongly dislike and more strongly befriend whom they consider as victims. To discard those hypotheses, supplementary analyses were performed to examine the interaction between students’ prosocial behavior and the interplay of dyadic perception of aggression and victimization with friendships and antipathies. Results indicated no effects of the individual prosocial behavior on the extent to which students befriend and dislike classmates whom they considered as aggressive or victimized (see details in Appendix B6).

## Discussion

Peer relationships play a central role in adolescents’ social development. Peer relationships might take positive forms, such as friendships (Bagwell and Smith 2011), but also negative forms, such as antipathies (Berger et al. 2011). Both types of relationships can be affected by how students perceive peers’ aggression and victimization. However, aggression and victimization may be evaluated and appreciated differently in classrooms depending on the extent that classrooms’ social norms sanction aggression, or promote and value prosocial behaviors. One way to change social norms is via educational interventions that can foster positive and more inclusive classroom environment.

This study examined whether the interplay of the dyadic perception of aggression and victimization with friendship and antipathy networks unfolds differently in classrooms that were part of a school-based intervention for promoting prosocial behaviors and civic engagement, using data from 530 Chilean seventh-grade students. A longitudinal social network approach was used to test the four hypotheses. In the models, the coevolution of aggression, victimization, and friendship or antipathies ties were modeled simultaneously controlling for network structural effects as well as the impact of prosocial behavior and sex.

It was expected that adolescents participating in this intervention would be less befriended by classmates who considered them as aggressors and more befriended by classmates who considered them as victims, compared to control classrooms. The effects of aggression and victimization on friendships were not significant in either intervention or control classrooms. An explanation for this finding might be that friendships, compared to antipathies, are more stable and permanent over time (Daniel et al. 2016; Hayes 1978). Therefore, it might be that prosocial interventions are more successful in ceasing antipathies than modifying friendships. Overall, positive classroom contexts seem to counteract the negative consequences of being disliked for aggressive and victimized students. Promoting prosocial behaviors across adolescence may reinforce a peer context in which externalizing (i.e., aggression) and internalizing (i.e., isolation) peer behaviors might be attenuated by the inclusive role of prosocial tendencies, where adolescents can support and cooperate with peers above and beyond their personal characteristics and their status in the peer network.

It also was anticipated that adolescents in intervention, compared to control classrooms, would be more disliked by classmates who considered them as aggressors and less disliked by classmates who considered them as victims. The findings indicate that in intervention classrooms adolescents who were considered as victims by peers were less likely to be disliked by those same peers. Similarly, compared to control classrooms, in intervention classrooms, adolescents who were considered as aggressive by peers were less likely to be disliked by those same peers. Even though this might seem counterintuitive since aggression should be more sanctioned in prosocial classrooms, it might be that in these classrooms sanctions to aggressive peers are not associated to social exclusion, but to other means, for example, by a decrease in social status instead of an increase in antipathy nominations. In other words, aggression may become less salient as a social asset in intervention classrooms. Together, the results show that the intervention was associated with classrooms in which perceived aggressors and victimized adolescents were less disliked. In this direction, educational interventions might be helpful in terms of reducing their involvement in antipathies, and consequently, its negative consequences. Positive peer contexts, including social support from peers, can serve a protective function, especially for victims (Storch et al. 2003). These results stress the importance of developing prosocial and empathic skills in schools.

One important feature of this study was the novel use of the dyadic perception networks, specifically about aggression and victimization. Previous research on peer relations (e.g., Dijkstra et al. 2012; Logis et al. 2013) has often treated aggression and victimization as individual characteristics by aggregating the dyadic information in proportion or standardized scores per student. However, this approach comes with the cost of losing the dyadic information (e.g., an aggression nomination of the student *i* over student *j*). Only recently, studies (Kisfalusi et al. 2019; Pál et al. 2016) have investigated the effect of the dyadic perception of disdain and respect on disliking and gossiping relationships, suggesting the importance of incorporating the dyadic perception on the study of peer relationships’ dynamics. Precisely, the combination of dyadic perception networks and multiplex social networks models represents an advance for modeling different types of networks (perceptions and relationships) simultaneously. This approach may open a promising area for further research that examines the effects of interventions on how perceptions of peers’ behaviors are associated with actual relationships to them.

This study has some limitations that should be considered. First, in this study, aggression, victimization, friendship and antipathy networks were constrained within school classes, as Chilean students spend most of their time in the same class. However, peer relationships may also occur at the grade or school level, and even outside school (Kerr et al. 2007), and particularly in the realm of problem behaviors (Kiesner et al. 2003, 2004). Future research can examine these various contexts (e.g., classroom, grade, school, and outside of school) providing a complete picture of the interplay of different types of peer relationships (Veenstra and Dijkstra 2011). Second, the fact that the maximum number of nominations were established on three could artificially limit the selection of classmates for the four types of networks, especially for friendships. There is evidence that the average number of friendship nominations per student tend to be higher than three (e.g., Gremmen et al. 2018; Laninga-Wijnen et al. 2018a, 2018b; Rulison et al. 2013) and also being larger in comparison to other types of networks such as antipathies, aggression, victimization, bullying, and defending (Daniel et al. 2016; Fujimoto et al. 2017; Huitsing et al. 2014, 2019).

Finally, due to the limited number of nominations, the focus of this study was limited to the interplay of the perception of aggression and victimization, and friendships and antipathies at the dyadic level. However, this interplay could also occur at both actor- and triadic-level. Future research should include these two levels by examining, for example, whether students less strongly dislike those who are generally considered as aggressor or victim, and whether friends tend to agree in their perception of a third classmate as an aggressor or victim.

## Conclusion

Both positive (e.g., friendships) and negative relationships (e.g., antipathies) can be affected by aggression and victimization, but also by how students perceive peers’ behaviors (Kisfalusi et al. 2019; Pál et al. 2016). The present study focuses on the associations between adolescents’ dyadic perceptions of peers’ aggression and victimization and peer relations, also considering how these associations differ in classroom contexts with different levels of prosocial norms. This study constitutes a methodological advance by combining the use of longitudinal multiplex social networks analysis with dyadic perception networks to examine the interplay of different types of adolescents’ relationships. The results indicate that dyadic perceptions of aggression and victimization have a significant effect on antipathies. This approach overcomes limitations of using aggregated scores on aggression and victimization based on peer nominations, acknowledging the particularity of dyadic perceptions and how these might affect the formation and maintenance of interpersonal ties. From an intervention perspective, these results evidence that educational interventions aimed at promoting prosocial behavior and civic engagement can play a significant role in how these perceptions are intertwined in adolescent peer dynamics. In this sense, prosocial interventions could protect students by fostering social settings in which antipathies are less associated with aggression and victimization at the dyadic level. This study provides insights for research-based intervention strategies designed to promote adolescents’ positive relationships in the classroom context.

## Appendix A1

**Table A1:** Descriptive classroom network information

Classroom	Type of classroom	*N*	% missing			Friendship average degree			Antipathy average degree			Aggression average degree			Victimization average degree		
			T1	T2	T3	T1	T2	T3	T1	T2	T3	T1	T2	T3	T1	T2	T3
1A	Intervention	47	0.15	0.14	0.18	0.49	2.82	2.91	2.54	2.82	2.89	2.44	2.82	2.89	2.34	2.80	2.71
1B	Intervention	50	0.07	0.05	0.07	2.24	2.66	2.45	2.26	2.66	2.45	2.05	2.60	2.41	1.90	2.58	2.23
2A*	Intervention	34	0.16	0.22	0.19	1.71	2.27	1.59	1.78	2.27	1.63	1.78	2.27	1.49	1.74	2.27	1.52
2B	Intervention	30	0.10	0.06	0.00	2.73	2.70	2.17	2.99	2.77	2.13	2.81	2.70	2.17	2.77	2.73	2.13
2C	Intervention	29	0.26	0.18	0.13	2.46	2.35	1.94	2.64	2.39	1.98	2.64	2.39	1.98	2.41	2.26	2.06
3A*	Intervention	48	0.02	0.02	0.08	2.49	2.81	2.57	2.49	2.81	2.46	2.44	2.81	2.37	2.47	2.76	2.55
4A	Intervention	35	0.06	0.03	0.02	2.66	2.73	2.43	2.78	2.73	2.46	2.75	2.73	2.40	2.48	2.70	2.22
4B	Intervention	34	0.00	0.05	0.00	2.85	2.83	2.47	2.91	2.82	2.47	2.71	2.79	2.35	2.56	2.63	2.21
4C	Intervention	31	0.00	0.00	0.00	2.90	2.74	2.45	2.77	2.65	2.00	2.74	2.74	2.13	2.39	2.52	1.74
5A	Control	43	0.11	0.11	0.16	2.60	2.57	2.34	2.65	2.57	2.34	2.57	2.65	2.34	2.63	2.55	2.34
6A	Control	40	0.23	0.23	0.17	2.54	2.62	2.18	2.54	2.62	2.18	2.46	2.42	2.12	2.47	2.55	2.18
6B	Control	39	0.18	0.18	0.11	2.99	3.00	2.42	2.99	3.00	2.34	2.95	2.97	2.37	2.89	2.97	2.37
7A	Control	50	0.14	0.14	0.18	2.26	2.30	2.33	2.28	2.30	2.33	2.21	2.30	2.28	2.30	2.23	2.23
7B*	Control	47	0.04	0.04	0.02	2.62	2.26	2.41	2.64	2.37	2.36	2.60	2.35	2.30	2.44	2.28	2.36
7C	Control	51	0.17	0.13	0.18	2.13	1.89	2.28	2.13	1.87	2.28	2.13	1.89	2.28	2.13	1.89	2.16
8A	Control	51	0.19	0.20	0.18	1.72	1.79	1.82	1.69	1.91	1.72	1.67	2.03	1.86	1.50	1.72	1.62
Av./Total	–	659	0.12	0.11	0.10	2.46	2.52	2.30	2.51	2.53	2.25	2.43	2.53	2.23	2.34	2.47	2.16

*N* the total number of students in the three measurement times

*Classrooms removed from the analyses

## Appendix A2

**Table A2:** Percentage of girls and average of prosocial behavior per classroom

Classroom	% of girls	Prosocial behavior time 1	Prosocial behavior time 2
1A	44	3.69	3.55
1B	49	3.54	3.63
2B	50	3.10	3.01
2C	48	3.17	3.14
4A	47	3.56	3.47
4B	50	3.57	3.41
4C	39	3.44	3.34
5A	58	3.55	3.49
6A	56	3.50	3.26
6B	68	3.52	3.51
7A	50	3.49	3.67
7C	64	3.60	3.53
8A	68	3.50	3.60
Average	53	3.48	3.43

## Appendix B1

**Missing data and composition change**

For the four types of networks, ordinary missing data were handled through the default RSiena procedure called last value carried forward method (Ripley et al. 2018) in which the impact of imputations on the results is minimized (Huisman and Steglich 2008). For each missing tie variable, the non-missing value (if any) is imputed; if the previous values are missing as well, the value 0 (referring to the absence of a tie) is assigned. Whenever imputed values are used, parameter estimate updates are based on the non-imputed parts of the data. Missing covariate data are, by default, replaced by the variable’s global mean.

To account for classroom composition changes (e.g., participants joining and leaving classrooms at the beginning or the end of the school year), structural zeros were specified for all ties toward and from participants who were absent at a given observation (Ripley et al. 2018).

## Appendix B2

**Meta-analytic procedure**

The bivariate estimations of the fifteen classrooms were summarized using a meta-analytic procedure with the *metafor* package in R (Viechtbauer 2010). This approach estimates and tests the mean as well as the standard deviation of each effect included in the model, using a method based on an iterated weighted least squares method and without making the assumption of a normal distribution (for more details, see Snijders and Baerveldt 2003). For each model (friendship and antipathy), two meta-analyses were performed; one for intervention classrooms, and other for control classrooms.

## Appendix B3

**Model specification**

The choice of the model parameters was based on a combination of three aspects: to control for structural networks effects (e.g., reciprocity, transitivity, balance) and relevant covariates (e.g., sex, prosocial behavior); to capture the interaction between networks adequately (e.g., the effect of one type of network on another type of network); and to keep the model parsimonious by assessing model convergence and goodness of fit. Specifically, three types of effects were included: structural network effects that model how the changes in each network depend on the network itself; cross-network effects that model how the changes in each network depend on the other network (e.g., antipathies depending on aggression); and covariate effects that model how changes in each network depend on actors’ attributes.

## Appendix B4

**Time heterogeneity tests**

By conducting time heterogeneity tests for each classroom, it was evaluated whether the effects’ estimates differed across the two periods. The overall test, including all the effects present in the models, indicated that time heterogeneity occurred only in a small subset of classrooms (three classrooms in the friendship model, and one classroom in the antipathy model). For this subset of classrooms, it was examined whether the cross-network effects (related to the four hypotheses) differed significantly across the two periods. Because the estimate for one cross-network effect (Aggression to Antipathy) in only one classroom differed significantly across periods, it was decided not to include additional effects in the model representing time heterogeneity.

## Appendix B5

**Goodness of fit**

The goodness of fit of the models was assessed by examining the extent to which the models explained additional features of the academic and friendship networks that were not explicitly included in the model specification. For the four types of networks, the distribution of outdegrees, indegrees, geodesic distance, and triad census were evaluated. The goodness of fit is assessed by comparing the Mahalanobis distance of the observations to the mean of the simulated values and computing the associated *p*-value (for more details, see Ripley et al. 2018). For the four statistics, the vast majority of the *p*-values for each type of classroom were between 0.10 and 0.90, indicating a good fit. The cases of unsatisfactory fit were associated with the outdegree distribution, in which the model slightly overrepresented the number of outgoing nominations with values of one and two, and underestimated the outdegrees with a value of three. An explanation for this poorer fit is that the number of outgoing nominations in each assessment point was limited to a maximum of three.

## Appendix B6

**Alternate models**

To examine whether adolescents with higher individual prosocial behavior would more strongly dislike and less strongly befriend whom they consider as aggressors, and less strongly dislike and more strongly befriend whom they consider as victims, two interaction effects were included in each model. For the friendship model, the *prosocial behavior* × *aggression to friendship*, and the *prosocial behavior* × *victimization to friendship* effects were included. For the antipathy model the *prosocial behavior* × *aggression to antipathy*, and the *prosocial behavior* × *victimization to antipathy* effects were included. The friendship and antipathy model were firstly estimated for each classroom, and then, the information was aggregated by conducting two meta-analyses (one for intervention and other for control classrooms).

In the case of the friendship model, because of the addition of these interaction effects, some classrooms presented convergence problems (i.e., low reliability of estimates). Accordingly, when necessary, one of the two interaction effects were fixed to the effect’s average of the rest of the classrooms of its type (intervention or control).

The results of the meta-analysis showed no significant effects for the two interaction effects in either intervention (Est._*pros. beh*. × *aggression to friendship*_ = 0.171, *p* = 0.844; Est._*pros. beh*. × *victimization to friendship*_ = −1.534, *p* = 0.273) or control classrooms (Est._*pros. beh*. × *aggression to friendship*_ = −0.486, *p* = 642; Est._*pros. beh*. × *victimization to friendship*_ = −0.935, *p* = 0.193). In the case of the antipathy model, all the classrooms presented good convergence indicators. No significant effects were found for the two interaction effects in either intervention (Est._*pros. beh*. × *aggression to antipathy*_ = −0.176, *p* = 0.454; Est._*pros. beh*. × *victimization to antipathy*_ = −0.212, *p* = 0.570) or control classrooms (Est._*pros. beh*. × *aggression to antipathy*_ = −0.174, *p* = 0.516; Est._*pros. beh*. × *victimization to antipathy*_ = 0.049, *p* = 0.888). Overall, these results suggest no effects of individual prosocial behavior on the extent to which aggressive and victimized adolescents are befriended and disliked.

## Footnotes

1 This test statistic results in a *z* score that under the null hypothesis of equal parameters has an approximating standard normal distribution (for more details see Ripley et al. 2018, p. 87).
